# Acoustic trauma slows AMPA receptor‐mediated EPSCs in the auditory brainstem, reducing GluA4 subunit expression as a mechanism to rescue binaural function

**DOI:** 10.1113/JP271929

**Published:** 2016-06-09

**Authors:** Nadia Pilati, Deborah M. Linley, Haresh Selvaskandan, Osvaldo Uchitel, Matthias H. Hennig, Cornelia Kopp‐Scheinpflug, Ian D. Forsythe

**Affiliations:** ^1^ Autifony Srl LaboratoriesMedicines Research Centre37135VeronaItaly; ^2^ MRC Toxicology Unit, Hodgkin BldgUniversity of LeicesterLeicesterLE1 9HNUK; ^3^ Department of Neuroscience, Psychology & BehaviourUniversity of LeicesterLeicesterLE1 9HNUK; ^4^ Instituto de Fisiología y Biología Molecular y Neurociencias, Universidad de Buenos Aires‐CONICET, Facultad de Ciencias Exactas y NaturalesCiudad UniversitariaC1428‐Buenos AiresArgentina; ^5^ Institute for Adaptive and Neural Computation, School of InformaticsUniversity of EdinburghEdinburghEH8 9ABUK; ^6^ SynthSysC. H. Waddington BuildingThe Kings Buildings CampusEdinburghUK; ^7^ Department of Biology IILudwig‐Maximilians‐Universität MünchenPlanegg‐MartinsriedD‐82152MunichGermany

## Abstract

**Key points:**

- Lateral superior olive (LSO) principal neurons receive AMPA receptor (AMPAR) ‐ and NMDA receptor (NMDAR)‐mediated EPSCs and glycinergic IPSCs.
- Both EPSCs and IPSCs have slow kinetics in prehearing animals, which during developmental maturation accelerate to sub‐millisecond decay time‐constants. This correlates with a change in glutamate and glycine receptor subunit composition quantified via mRNA levels.
- The NMDAR‐EPSCs accelerate over development to achieve decay time‐constants of 2.5 ms. This is the fastest NMDAR‐mediated EPSC reported.
- Acoustic trauma (AT, loud sounds) slow AMPAR‐EPSC decay times, increasing GluA1 and decreasing GluA4 mRNA.
- Modelling of interaural intensity difference suggests that the increased EPSC duration after AT shifts interaural level difference to the right and compensates for hearing loss.
- Two months after AT the EPSC decay times recovered to control values.
- Synaptic transmission in the LSO matures by postnatal day 20, with EPSCs and IPSCs having fast kinetics. AT changes the AMPAR subunits expressed and slows the EPSC time‐course at synapses in the central auditory system.

**Abstract:**

Damaging levels of sound (acoustic trauma, AT) diminish peripheral synapses, but what is the impact on the central auditory pathway? Developmental maturation of synaptic function and hearing were characterized in the mouse lateral superior olive (LSO) from postnatal day 7 (P7) to P96 using voltage‐clamp and auditory brainstem responses. IPSCs and EPSCs show rapid acceleration during development, so that decay kinetics converge to similar sub‐millisecond time‐constants (τ, 0.87 ± 0.11 and 0.77 ± 0.08 ms, respectively) in adult mice. This correlated with LSO mRNA levels for glycinergic and glutamatergic ionotropic receptor subunits, confirming a switch from Glyα2 to Glyα1 for IPSCs and increased expression of GluA3 and GluA4 subunits for EPSCs. The NMDA receptor (NMDAR)‐EPSC decay τ accelerated from >40 ms in prehearing animals to 2.6 ± 0.4 ms in adults, as GluN2C expression increased. *In vivo* induction of AT at around P20 disrupted IPSC and EPSC integration in the LSO, so that 1 week later the AMPA receptor (AMPAR)‐EPSC decay was slowed and mRNA for GluA1 increased while GluA4 decreased. In contrast, GlyR IPSC and NMDAR‐EPSC decay times were unchanged. Computational modelling confirmed that matched IPSC and EPSC kinetics are required to generate mature interaural level difference functions, and that longer‐lasting EPSCs compensate to maintain binaural function with raised auditory thresholds after AT. We conclude that LSO excitatory and inhibitory synaptic drive matures to identical time‐courses, that AT changes synaptic AMPARs by expression of subunits with slow kinetics (which recover over 2 months) and that loud sounds reversibly modify excitatory synapses in the brain, changing synaptic function for several weeks after exposure.

## Key points

- Lateral superior olive (LSO) principal neurons receive AMPA receptor (AMPAR) ‐ and NMDA receptor (NMDAR)‐mediated EPSCs and glycinergic IPSCs.
- Both EPSCs and IPSCs have slow kinetics in prehearing animals, which during developmental maturation accelerate to sub‐millisecond decay time‐constants. This correlates with a change in glutamate and glycine receptor subunit composition quantified via mRNA levels.
- The NMDAR‐EPSCs accelerate over development to achieve decay time‐constants of 2.5 ms. This is the fastest NMDAR‐mediated EPSC reported.
- Acoustic trauma (AT, loud sounds) slow AMPAR‐EPSC decay times, increasing GluA1 and decreasing GluA4 mRNA.
- Modelling of interaural intensity difference suggests that the increased EPSC duration after AT shifts interaural level difference to the right and compensates for hearing loss.
- Two months after AT the EPSC decay times recovered to control values.
- Synaptic transmission in the LSO matures by postnatal day 20, with EPSCs and IPSCs having fast kinetics. AT changes the AMPAR subunits expressed and slows the EPSC time‐course at synapses in the central auditory system.

**Abbreviations**
d‐AP5: d‐2‐amino‐5‐phosphonopentanoic acid
ABR: auditory brainstem response
AT: acoustic trauma
aVCN: anterior ventral cochlear nucleus
CNQX: 6‐cyano‐7‐nitroquinoxaline‐2,3‐dione
GluA1‐4: glutamate receptor subunit, AMPA subtype: 1–4.
GluN1‐2: glutamate receptor subunit, NMDA subtype: 1, 2a–d.
Glyα1‐4: glycine receptor alpha subunit 1–4
GlyR: glycine receptor ion channel
IID: interaural intensity difference
LSO: lateral superior olive
NBQX: 2,3‐dioxo‐6‐nitro‐1,2,3,4‐tetrahydrobenzo[*f*]quinoxaline‐7‐sulfonamide
MNTB: medial nucleus of the trapezoid body
MSO: medial superior olive
qRT‐PCR: quantitative reverse transcription PCR
SOC: superior olivary complex
SPL: sound pressure level
Tau, τ: EPSC decay time‐constant

## Introduction

Synapses undergo multiple forms of activity‐dependent refinement during development, including synaptic scaling, competition/elimination and adaptation of synaptic current time‐course through changes in the subunit composition of channels at the synapse. It is well established that exposure to damaging volumes of sound (acoustic trauma, AT) raises auditory thresholds, injures cochlear hair cells and afferent synapses, and causes hearing loss. The extent to which AT damages or changes aspects of the central auditory pathway is often difficult to assess because effects in the cochlea inevitably propagate into the brain. The objective of this study was to determine the extent to which AT might induce changes at central excitatory and inhibitory synapses of the superior olivary complex (SOC) and to investigate whether this affects the interaural level computation for sound localization.

Neurons in the lateral superior olive (LSO) of the SOC are amongst the first to receive inputs from both ears. Excitatory (glutamatergic) inputs from the ipsilateral cochlear nucleus are integrated with inputs from the contralateral cochlear nucleus via an inhibitory (glycinergic) projection from the medial nucleus of the trapezoid body (MNTB) (Goldberg & Brown, 1968; Glendenning *et al*. 1985; Tsuchitani, 1988; Wu & Kelly, 1995; Kopp‐Scheinpflug *et al*. 2011). Neuronal, intrinsic properties (Barnes‐Davies *et al*. 2004) and synaptic inputs are tonotopically organized (Rietzel & Friauf, 1998) so that contralateral projections correspond to ipsilateral projections. Hence LSO principal neurons integrate excitatory and inhibitory synaptic responses to the same sound frequencies from opposite ears, and compute interaural intensity differences (IIDs) to localize sound across the azimuth (Tollin & Yin, 2002; Tollin, 2003).

Both excitatory and inhibitory inputs to the LSO undergo synaptic reorganization before hearing onset at postnatal day 11/12 (Kandler *et al*. 2009). Notably, chloride gradients evolve, such that a mixed glycine‐ and GABA‐mediated synaptic response shifts from depolarizing to hyperpolarizing (Kim & Kandler, 2003, 2010; Gillespie *et al*. 2005). After hearing onset IPSCs are further refined (Sanes & Friauf, 2000; Kandler & Gillespie, 2005; Kandler *et al*. 2009): there is a tonotopic reorganization (Kandler *et al*. 2009) driven by spontaneous activity (Clause *et al*. 2014), a reduction in the number of terminal arbors (Sanes & Siverls, 1991; Sanes & Takacs, 1993) and an acceleration of IPSC time‐courses (Kandler & Friauf, 1995; Walcher *et al*. 2011). This is accompanied by a shift in neurotransmission of the IPSC from mixed GABA/glycine to predominantly glycinergic receptors by P14 (Nabekura *et al*. 2004; Sterenberg *et al*. 2010) although GABA spillover to presynaptic MNTB axons in the LSO persists beyond P14 (Weisz *et al*. 2016).

Information transmission along the auditory nerve is compromised and centrally compensated following sound‐induced trauma (Kujawa & Liberman, 2009) but we postulate that other activity‐dependent modulatory changes in the CNS can further influence auditory processing; for example, by changes in the subunit composition of the synaptic receptors. Such changes would change channel open time, and so be reflected in the decay kinetics of synaptic currents (Magleby & Stevens, 1972; Raman *et al*. 1994; Koike‐Tani *et al*. 2005) thereby modifying synaptic integration and neuronal output (Johnston *et al*. 2009). Thus, by examining the kinetics of synaptic currents and comparing this to subunit expression, we can gain insights into activity‐dependent mechanisms affecting auditory processing.

Here we characterize the development of both components of the IID computation in the LSO; measuring excitatory (E) and inhibitory (I) synaptic currents across an age range that spans postnatal maturation in four age groups (see Fig. 2 *A*): Pre‐hearing, Hearing onset, Juvenile and Young adult mice. Then having determined the developmental time‐course of the synaptic currents, we demonstrate that over‐excitation in the form of AT changes the expression of receptor subunit mRNA, modifies receptor composition and so adapts the synaptic kinetics. The results show that AT causes activity‐dependent changes in synaptic AMPA receptor (AMPAR) subunits, slowing fast excitatory synaptic currents for weeks and modifying IID processing in the LSO.

## Methods

We have employed whole‐cell patch clamp methods to characterize synaptic currents and quantitative PCR to examine subunit expression over the developmental time‐course. Then we have used *in vivo* induction of sound trauma and confirmation of hearing deficits by auditory brainstem responses (ABRs), combined with *in vitro* electrophysiological assessment of central synaptic function in the LSO in experiments that conform to the principles of UK regulations relating to the use of animals in research (Grundy, 2015). The impact of these changes on IID processing was assessed using computational modelling.

### Preparation of brain slices

Male and female *CBA/Ca* mice (aged 6–96 days) were killed by decapitation (using methods approved by the University of Leicester Ethical Committee and the Animals, Scientific Procedures, Act 1986, UK) and brainstem slices containing the SOC were prepared as described previously (Barnes‐Davies & Forsythe, 1995; Johnston *et al*. 2008). Coronal slices (150–200 μm thickness) of SOC containing the LSO were cut in a low‐sodium artificial cerebrospinal fluid (aCSF) at 0°C. Slices were then incubated in normal aCSF at 37°C for 1 h and subsequently stored at room temperature. The composition of the normal aCSF was (in mm): 125 NaCl, 2.5 KCl, 26 NaHCO_3_, 10 glucose, 1.25 NaH_2_PO_4_, 2 sodium pyruvate, 3 *myo*‐inositol, 2 CaCl_2_, 1 MgCl_2_ and 0.5 ascorbic acid. The pH was 7.4 when bubbled with 95% O_2_/5% CO_2_. The dissection was performed in low‐sodium aCSF, with 250 mm sucrose substituted for NaCl, and CaCl_2_ and MgCl_2_ concentrations adjusted to 0.1 and 4 mm, respectively.

### Electrophysiology

**Figure 1:**
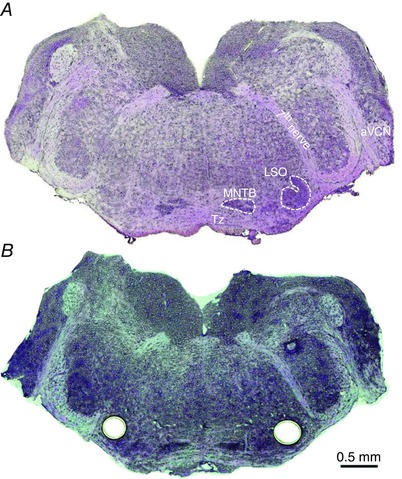
**Removal of tissue from cryostat sections of brainstem nuclei for mRNA extraction** Tissue was collected from cryostat‐sectioned brainstems under visual inspection. *A*, a transverse brainstem section (20 μm thick) was briefly fixed with ethanol and stained with cresyl violet. The trapezoid body (Tz), medial nucleus of the trapezoid body (MNTB), lateral superior olive (LSO) and the anterior ventral cochlear nucleus (aVCN) are labelled. The 7th nerve and the trapezoid body are also indicated. *B*, a similarly treated brainstem section after bilateral removal of the LSO using Zeiss PALM MicroBeam laser dissection.

One slice at a time was transferred to a temperature‐controlled experimental chamber (Model 7800, Campden Instruments, Loughborough, UK) mounted on the stage of an upright microscope. The chamber was perfused at 1 ml min^−1^ with normal aCSF at 36°C (gassed with 5% CO_2_/95% O_2_). All electrophysiology experiments were conducted at 36°C. LSO neurons were visualized using differential interference contrast (DIC) optics (MicroInstruments, Long Hanborough, UK) and a 40× water‐immersion objective (Zeiss, Oberkochen, Germany). Whole‐cell patch‐clamp recordings were made from LSO principal neurons using an Axopatch 200B amplifier, Digidata 1440 and pCLAMP10 software (Molecular Devices, Wokingham, UK). Data were sampled at 20–50 kHz and filtered at 5 kHz. Patch pipettes were made using thin‐walled borosilicate glass (GC150F7.5, Harvard Apparatus, Edenbridge, UK) and a two‐stage vertical puller (PC‐10, Narishige, London, UK) and filled with (in mm): potassium gluconate 97.5; KCl 32.5; EGTA 5.0; Hepes 40; MgCl_2_ 1; Na_2_phosphocreatine 5 (adjusted to pH 7.2 with KOH). Voltage signals were not corrected for the liquid junction potential (−11 mV). For NMDA receptor (NMDAR)‐EPSC recordings, CsCl was substituted for KCl and potassium gluconate. Whole‐cell capacitance and series resistances were noted from the amplifier and 70% series resistance compensation was used. A bipolar platinum electrode was positioned across the MNTB or over the ipsilateral fibres from the ventral cochlear nucleus (Figs 1 and 3 *A*) and connected to a voltage stimulator (DS2A, Digitimer Ltd, Welwyn Garden City, UK). Stimuli (200 μs duration) were delivered at 0.25 Hz with an intensity to give maximal postsynaptic responses. Glycinergic IPSCs were recorded in the presence of 10 μm bicuculline, 10 μm 6‐cyano‐7‐nitroquinoxaline‐2,3‐dione (CNQX) or 2,3‐dioxo‐6‐nitro‐1,2,3,4‐tetrahydrobenzo[f]quinoxaline‐7‐sulfonamide (NBQX) and 20 μm d‐2‐amino‐5‐phosphonopentanoic acid (d‐AP5) whereas AMPAR‐mediated EPSCs were recorded in the presence of 10 μm bicuculline, 0.5–1 μm strychnine and 20 μm d‐AP5. NMDAR‐mediated EPSCs were recorded in the absence of d‐AP5 and pharmacologically isolated by perfusion with 10 μm bicuculline, 1 μm strychnine and 10 μm NBQX. Miniature EPSCs (mEPSCs) and mIPSCs were recorded in the presence of TTX (0.5 μm) and the respective blockers for inhibitory or excitatory synaptic transmission (as detailed above). All chemicals and drugs were obtained from Sigma (Gillingham, UK), except bicuculline and d‐AP5, which were from Tocris (Bristol, UK). Values of *n* refer to the number of neurons from which recordings were made and sample groups comprised data collected from at least three animals. IPSC and EPSC decay times and amplitudes were measured from averaged traces (10–15 records). mIPSC and mEPSC decay times were measured from averaged traces (20 records). Excitatory and inhibitory synaptic events were recorded across the full extent of the LSO and no tonotopic relationship was apparent (data not shown).

### Recording of ABRs

*CBA/Ca* mice (P7–96) were anaesthetized intraperitoneally with a combination of fentanyl (0.15 mg kg^−1^), fluanisone (5 mg kg^−1^) and hypnovel (2.5 mg kg^−1^). ABRs were evoked by tone pips (at 8/12/16/24/30 kHz, 1 ms rise and fall times, 5 ms duration) or clicks (broadband between 2 and 20 kHz, 100 μs), which were produced by a Thurlby Thandar arbitrary waveform generator (TGA 1230, 300 MHz, Tucker Davis, Alachua, FL, USA) and applied at 10 Hz in free field unilaterally using a B&K microphone (B&K 4192). The final ABR constituted an average of 100–400 individual traces recorded by intradermal electrodes (positive, negative and ground electrodes were inserted subcutaneously at the vertex, mastoid and back, respectively) with an input gain of 5000 connected to an amplifier (Medelc Sapphire 2A, Oxford Instruments, Oxford, UK) and sampled at 16 kHz. Hearing thresholds were determined by attenuating the initial stimulus intensity [clipped at 94 dB sound pressure level (SPL)] by 10 or 3 dB SPL steps (Tucker Davis) until ABR waves I and II could no longer be defined. The wave amplitude was measured as the absolute value of the peak (positive or negative) from 0 μV. Each wave represents the synchronous activity of sequential nuclei in the ascending auditory pathway. In mice the SOC output provides a major contribution to the formation of ABR wave IV, as defined with respect to the Jackson Laboratory phenotyping protocol: (http://phenome.jax.org/db/q?rtn=projects/docstatic&doc=Jaxpheno8/Jaxpheno8_Protocol#Procedure_ABR).

The sample size (*n*) indicates the number of animals from which an ABR was measured at the specified frequency.

### Acoustic trauma

Male or female *CBA/Ca* mice were anaesthetized as above and placed for 2 h in a custom‐made sound‐insulated box containing a loudspeaker [Prosound WF09K, frequency range 4–40 kHz that delivered a broadband noise (0–30 kHz) at 110 dB SPL]. Animals were exposed once bilaterally; the loudspeaker was located above the head at a distance of around 4 cm. Control animals (shams) of the similar age as the AT animals were anaesthetized with the same procedure but were not exposed to sound.

### Statistical analysis of electrophysiology data

Data were analysed using the software package GraphPad Prism 6. Data are plotted as mean ± SEM, with *n* being specified as follows: voltage‐clamp, *n* = neurons recorded in tissue from at least three animals; ABR, *n* = animal. Datasets were first tested for equality of variance, and the appropriate statistical test then applied. Independent *t* tests were used when comparing two groups (with Welch's correction in cases of unequal variance). When comparing three or more groups, ANOVA (one‐way or two‐way) was used with *post hoc* analysis (one‐way, Tukey; two‐way, Sidak's multiple comparisons). In cases of unequal variance, a Kruskall–Wallis test was employed. For comparisons of cumulative distribution, a Kolmorogov–Smirnov (K‐S) test was used. Statistical significance was concluded when *P *≤ 0.05.

### RNA extraction and quantitative reverse transcriptase PCR analysis

**Figure 2:**
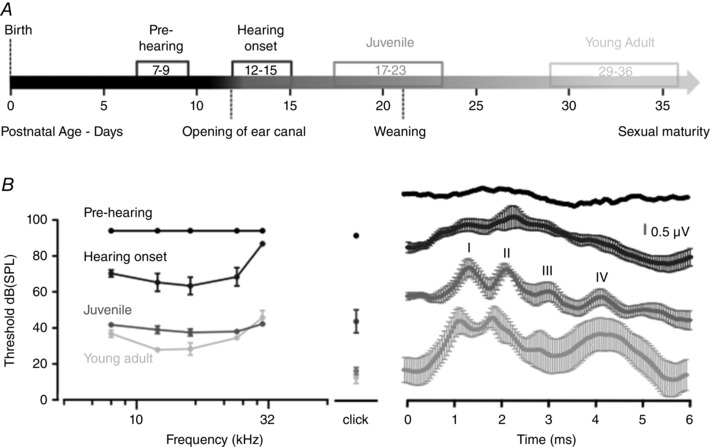
**Auditory brainstem responses (ABRs) reach maturity about 2 weeks after Hearing onset in CBA/Ca mice** *A*, ABR measurements were conducted in mice from four age groups: Pre‐hearing (P7–9, *n* = 4–5), Hearing onset (P12–15, *n* = 7–8), Juvenile (P17–23, *n* = 21–29) and Young adult (P29–36, *n* = 5–8). *B*, ABRs were obtained and average response thresholds (mean ± SEM) were plotted for five frequencies between 8 to 30 kHz and for a click stimulus. Right: averaged ABR waves (± SEM) for each age group are shown in response to 24 kHz tones at 94 dB SPL; waves I–IV are indicated on ABRs. Statistical comparison was performed by a two‐way ANOVA (8–30 kHz frequencies) and one‐way ANOVA (click stimuli). There was a significant decrease in auditory thresholds from Pre‐hearing to Juvenile mice (*P* < 0.0001) for all frequencies (except at 30 kHz for Pre‐hearing to Hearing onset, *P* = 0.5035). There was no significant improvement in auditory thresholds from Juvenile to Young adult, except at 12 kHz (*P* = 0.022).

For developmental studies, male and female CBA/Ca mice were used within the same age groups as specified in Fig. 2 *A*. For studies following AT, male and female CBA/Ca mice aged P23–29 were used (noise exposure occurred 7 days previously). After killing (as above) the brainstem was dissected into ice‐cold PBS, transferred to OCT medium and frozen using dry ice and hexane. Transverse cryostat sections (20 μm) were taken from the unfixed tissue and mounted onto Zeiss PEN‐Membrane covered slides. Sections were fixed with ethanol and stained with cresyl violet. The LSO was dissected from each section using a Zeiss PALM MicroBeam laser dissection microscope (Fig. 1) and the RNA was extracted. Ten sections were used from each animal. Total RNA was isolated using the QIAGEN RNeasy protocol (Applied Biosystems, Foster City, CA, USA). cDNA synthesis was performed using Superscript III (Invitrogen). The mRNA levels for GlyR, AMPAR and NMDAR subunits were measured from the same samples. PCR primers were designed (Table 1) using the Roche Applied Sciences Primer Design tool (http://www.roche‐applied‐science.com) and purchased from Invitrogen. Primer sequences (Invitrogen) were optimized and γ‐actin (Grant *et al*. 2006) or Rpl‐44 were used as housekeeping genes. The GluA primers were specifically selected to detect both flip and flop isoforms (and did not distinguish between them).

**Table 1:** PCR primers

Subunit	Forward primer	Reverse primer
GluA1	CCAATTTCCCCAACAATATCC	AAAGCTGTCGCTGATGTTCA
GluA2	CAGTTTCGCAGTCACCAATG	ACCCAAAAATCGCATAGACG
GluA3	CCACTTGGATTCCTCCAATAGT	GCATACACCCCTCTGGAGAA
GluA4	CTGCCAACAGTTTTGCTGTG	AAATGGCAAACACCCCTCTA
GlyR1	CGATTCTACCTTTGGGAGACC	TTCAGCCTCTTTGGAAGCA
GlyR2	GACTACACAGAGTTCAGGTTCCAG	TCCAGATGTCAATTGCTTTCA
GlyR3	GGGAAGCCGCACTGTTACT	GAGATCGCGCACTGTTTGT
GlyR4	CTGCCAACAGTTTTGCTGTG	GCCAGACGTGGGTCATTC
GluN1A	TGAGTCCAAGGCAGAGAAGG	CGCTTGCAGAAAGGATGATG
GluN2A	ATTCAACCAGAGGGGCGTA	TTCAAGACAGCTGCGTCATAG
GluN2B	GGGTTACAACCGGTGCCTA	CTTTGCCGATGGTGAAAGAT
GluN2C	GAAGCGGGCCATAGACCT	TGGCAGATCCCTGAGAGC
GluN2D	TGCGATACAACCAGCCAAG	AGATGAAGGCGTCCAGTTTC
γ‐Actin	CCCTAGCACCTAGCACGATGA	GCCACCGATCCAACTGAGTAC

Primers were verified by a BLAST sequence analysis and gel electrophoresis. Quantitative reverse transcriptase PCR (qRT‐PCR) was performed using SYBR Green PCR Master Mix in the ABI PRISM 7700 Sequence Detection System (Applied Biosystems). The thermal cycler protocol was: stage 1, 50°C for 2 min; stage 2, 95°C for 10 min; and stage 3, 40 cycles at 95°C for 15 s and 60°C for 1 min. Each sample was run in triplicate. Quantification was performed using the method of Pfaffl (2001).

### Statistical analysis of qRT‐PCR data

Data are presented as means ± SEM (‘*n*’ constituted data from one animal). Data were analysed using the statistical software SPSS Statistics 22. The equality of variance was first established from each data set. In cases of equal variance, independent *t* tests were used to compare two groups. For three or more comparisons a one‐way ANOVA with Tukey *post hoc* testing was employed. In cases where the assumption of equal variance was violated, a Mann–Whitney U test was used and Welch's test applied.

### Computational modelling

The LSO neuron model used here is based on a previous implementation by Karcz *et al*. (2011), with parameters set to match the new *in vitro* data obtained in this study at different ages or following acoustic trauma (Table 2). The neuron was implemented as a single‐compartment, leaky integrate and fire unit with conductance‐based synapses:

$$\begin{array}{ccc} \tau_{m}dV\;\left(t\right)/dt & = & \sum^{g_{i}}\;\left(t\right)V\left(t\right)+\sum^{g_{c}}\left(t\right)\left(V\left(t\right)-E_{c}\right)\\ & & -\;(V\left(t\right)-V_{r}) \end{array}$$

**Table 2:** Parameters used in the LSO neuron simulations

	τ_i_(ms)	τ_c_(ms)	$g_{i}^{m}$	$g_{c}^{m}$	*V*_th_(mV)
Hearing onset	0.73	1.55	3.8	14.8	−50.6
Young adult	0.76	0.87	7.5	4.4	−37.2
After AT	1.23	0.83	6.4	5.8	−37.2

τ_i_ = Ipsilateral decay tau (ms); τ_c_ = contralateral decay tau (ms); $g_{i}^{m}$ = ipsilateral peak conductance; $g_{c}^{m}$ = contralateral peak conductance; *V*_th_ = membrane potential threshold of spike (mV).

The membrane time‐constant (τ_m_) was 1.5 ms (Wu & Kelly, 1991; Sanes & Takacs, 1993), and the resting potential *V*_r_ was −60 mV. The neuron received four ipsilateral excitatory and four contralateral inhibitory inputs, with total conductances *g*_i_ and *g*_c_ and associated reversal potentials *E*_i_ = 0 mV and *E*_c_ = −75 mV (Balakrishnan *et al*. 2003). Synaptic conductances are expressed in units of the leak, and are therefore dimensionless, and were modelled as:

$$\tau_{i,c}dg_{\left\{i,c\right\}}\left(t\right)/dt=g_{\left\{i,c\right\}}^{m}\;\delta \left(t-t_{s}\right)-g_{i,c}\left(t\right),$$

with spike times {*t*_s_}, time‐constants τ_i_ and τ_c_ and peak conductances *g*_i_ and *g*_c_ for the ipsi‐ and contralateral inputs, respectively, which were set to match experimental data in this study (Table 2). The neuron produces a spike when the membrane potential exceeds the threshold *V*_th_, after which it is reset to *V*_reset_ = −75 mV.

Input spike trains were generated, as described by Karcz *et al*. (2011), to reproduce the level of precision previously reported for sound‐evoked responses in ventral cochlear nucleus (VCN) and MNTB neurons of mice (Kopp‐Scheinpflug *et al*. 2003). Noise was introduced by shifting each spike, and all successive spikes, by a random amount drawn from an exponential distribution with mean and standard deviation σ_i_ = 0.15 ms and σ_c_ = 0.4 ms. The mean latency of the contralateral inputs was set to Δ_c_ = 1.4 ms (Karcz *et al*. 2011). Sound level‐dependent latency differences in MNTB neurons were included by increasing the ipsilateral latency by:

δl=−1.25 ms Rc/Ri−1,

where *R*_i_ and *R*_c_ are the ipsi‐ and contralateral firing rates, respectively (FitzGerald *et al*. 2001; Kopp‐Scheinpflug *et al*. 2003; Karcz *et al*. 2011). Tuning curves were simulated by fixing the spike rate at the ipsilateral input, while systematically changing the rate of the contralateral input from 0 to twice that of the ipsilateral input. Note that these tuning curves do not necessarily represent true IID tuning curves because the precise relationship between SPL and VCN response was not taken into account here. In contrast to Karcz *et al*. (2011), the optimal tuning curve was estimated for each condition, by setting the synaptic parameters to match the *in vitro* data in this study, and adjusting the spike threshold such that the Fisher information estimated from the simulated IID tuning curve was maximized. Fisher information was computed for each IID level *x* as:

$$F\;\left(x\right)=\;r^{\prime }{\left(x\right)}^{2}/s{\left(x\right)}^{2}$$

where *r*′(*x*) is the mean response, differentiated with respect to IID, and *s*(*x*) the standard deviation of the response (Dean *et al*. 2005). The mean response was estimated by fitting a sigmoidal function to the simulated IID tuning curve, and noise by fitting a Gaussian function to the response standard deviation, both using 40 different realizations of the same IID tuning curve. Only the values of Fisher information within a range of ± 25% of equal amplitudes at the ipsi‐ and contralateral inputs were considered for optimizing the spike threshold, as a lack of response variability at the flanks of the tuning curves could lead to inflated values. Fisher information was computed for a range of physiologically plausible firing thresholds, and the threshold which gave, on average, the best performance across a range of ipsilateral input firing frequencies from 100 to 300 Hz were selected. This optimization was done for all ages, but not for the simulations following AT; here we assume that at least in the short term thresholds remained at the level of a mature animal.

## Results

We first assessed the time‐course of maturation of normal auditory function *in vivo* by measuring ABRs and then related this to synaptic time‐course in the LSO, by using whole‐cell patch recording from brainstem slices. Developmental changes in LSO mRNA for excitatory and inhibitory receptor‐ion channel subunits were also assessed by qRT‐PCR on tissue isolated by laser microdissection. Induction of AT caused elevated ABR thresholds in Juvenile mice, slowed excitatory synaptic current decays and changed receptor‐ion channel subunit mRNA levels, while the impact of these changes was interpreted through modelling of these changes on interaural intensity difference.

### Maturation of auditory function

A developmental time‐course for the ABR hearing threshold was determined in CBA/Ca mice by assessing four age groups (Fig. 2 *A*): Prehearing (P7–9), Hearing onset (P12–15), Juvenile (P17–23) and Young adult (P29–36). Prehearing and Hearing onset mice showed higher ABR thresholds and poorly defined ABR waves (Fig. 2 *B*) compared to the two older age groups, which showed normal ABRs and thresholds (Fig. 2 *B*). There was a minor further improvement in the ABR thresholds at mid‐frequencies (12 kHz) on maturation from Juveniles to Young adult.

### Acceleration and convergence of EPSC and IPSC kinetics with maturation

**Figure 3:**
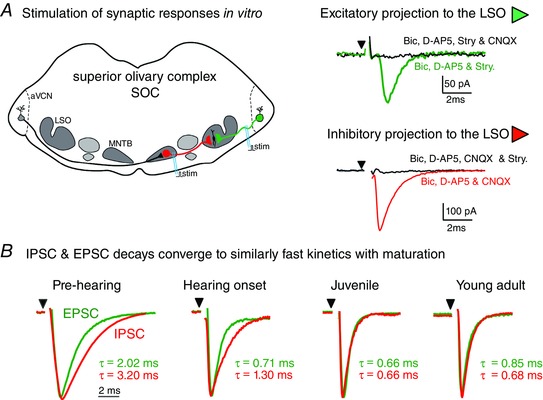
**EPSCs and IPSCs undergo refinement during development until identical time‐courses are generated** *A*, diagram of the *in vitro* brain slice showing the location of the LSO, other nuclei and stimulation sites in the superior olivary complex. Ipsilateral excitatory inputs from the aVCN to the LSO are highlighted in green and contralateral inhibitory inputs from the MNTB are highlighted in red. Bipolar stimulating electrodes were placed over the MNTB and over the axons from the ipsilateral cochlear nucleus, as indicated. EPSCs (green, upper right inset) were recorded in the presence of bicuculline (10 μm), strychnine (0.5 μm) and d‐AP5 (20 μm) and were blocked by the application of CNQX (10 μm). IPSCs (red, lower right inset) were recorded in the presence of bicuculline (10 μm), CNQX (10 μm) and d‐AP5 (20 μm) and were blocked by the application of strychnine (0.5 μm). *B*, representative examples of EPSCs (green) and IPSCs (red) recorded in Pre‐hearing (P6), Hearing onset (P13), Juvenile (P22) and Young adult (P33) age groups. Each example is an average (*n* = 20) and EPSCs and IPSCs are superimposed with respect to the rising phase and normalized to the peak amplitude; the stimulation is indicated by the black arrowhead and stimulus artefacts have been removed for clarity. The decay time‐constant (τ) for each trace is indicated. All recordings were from neurons held at a potential of −70 mV.

The kinetics of excitatory and inhibitory synaptic inputs to the LSO measured *in vitro* using whole‐cell patch recording (Fig. 3 *A*) show a characteristic acceleration over a similar developmental time‐course to that described for ABR thresholds *in vivo*.

**Figure 4:**
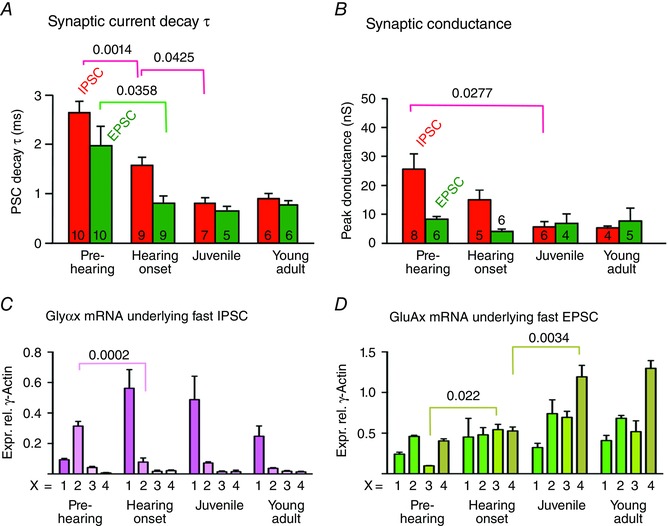
**Mean data showing acceleration in synaptic decay and reduced conductance with changes in the subunit mRNA levels across development** *A*, bar charts summarizing EPSC (green, mean ± SEM) and IPSC (red) decay τ. *B*, conductance for the four age groups of mice used in this study. Sample size (*n*) is indicated in the respective bar. EPSCs and IPSCs converge to similar time‐course and conductance in Juvenile and Young adult mice. Significant changes are indicated by the line and *P* value. *C*, plot of the GlyR subunit mRNA expression (x = Glyα1–4) relative to γ‐actin in the LSO for the four age groups used in this study (*n* = 3), showing the early switch from Glyα2 to Glyα1 as significant, as indicated by the lines and *P* values. *D*, glutamate receptor subunit mRNA expression (x = GluA1–4) for the four age groups (*n* = 3). There are significant increases in GluA3 from Pre‐hearing to Hearing onset and for GluA4 on maturation from Hearing onset to Juvenile. Significance of changes between age groups was assessed by one‐way ANOVA and indicated as values of *P* between the respective data.

Evoked glycinergic IPSCs exhibited a developmental acceleration that stabilized after Hearing onset (from around P18), with a decay time‐constant (τ) accelerating from 2.6 ± 0.2 ms (mean ± SEM, *n* = 10, Pre‐hearing) to 0.79 ± 0.1 ms (*n* = 7, Young adult; Figs 3 *B* and 4 *A*, red traces). In parallel, the maximal evoked IPSC conductance progressively declined from 25.3 ± 5.3 nS (*n* = 8) in Pre‐hearing mice to 5.4 ± 1.7 nS (*n* = 6) in the Juvenile mice (Fig. 4 *B*, red) consistent with previous reports measuring refinement of inhibitory synaptic inputs to the LSO (Kim & Kandler, 2003). These synaptic parameters were then stable as mice matured in the Young adult group. IPSC rise time also exhibited developmental acceleration, from 0.83 ± 0.08 ms (*n* = 9) in Pre‐hearing mice to 0.48 ± 0.03 ms (*n* = 9) in Hearing onset mice, with no significant change thereafter.

The decay kinetics of EPSCs decreased from 1.97 ± 0.39 ms (*n* = 10) in Pre‐hearing mice to 0.65 ± 0.09 ms (*n* = 5) in Juveniles (green traces, Figs 3 *B* and 4 *A*). In parallel, EPSC rise time decreased from 1.18 ± 0.16 ms (*n* = 8) in Pre‐hearing mice to 0.47 ± 0.69 ms (*n* = 5) in Juvenile mice; while the amplitude of the maximal evoked EPSC conductance remained stable through development from Pre‐hearing to Young adult (Fig. 4 *B*, green). The EPSC and IPSC kinetics converged to near identical sub‐millisecond values in the mature auditory system with decay time‐constants of 0.65 ± 0.09 ms (*n* = 5) and 0.79 ± 0.11 ms (*n* = 7) respectively, for Juveniles and 0.77 ± 0.08 ms (*n* = 6) and 0.87 ± 0.11 ms (*n* = 6) respectively, for Young adults. This convergence is illustrated by superimposed EPSCs and IPSCs for each of the age groups used in this study (Fig. 3 *B*) and consistent with a binaural physiological mechanism of IID detection through integration of excitatory and inhibitory responses from ipsilateral and contralateral ears, respectively.

This acceleration in the synaptic decay kinetics with development is consistent with functional changes in the subunit composition of AMPAR and/or glycine receptors during maturation. We used qRT‐PCR to test for changes in mRNA levels of glycine receptor (GlyR) and AMPAR subunits in LSO tissue from mice aged P9, P14, P22 and P35 (Fig. 4 *C*, *D*). The mRNA levels of GlyR subunits (relative to the housekeeping gene) showed a dramatic increase in Glyα1 over Glyα2 at Hearing onset, which stabilized or declined slightly into adulthood. This well‐established switch in GlyR subunit expression over development, with an early predominance of Glyα2 in Pre‐hearing immature animals, (Fig. 4 *C*) and a switch to Glyα1 dominance on maturation after Hearing onset, has been reported previously in the spinal cord (Takahashi *et al*. 1992) and the SOC (Piechotta *et al*. 2001). The switch to dominance of GlyR1 mRNA preceded the acceleration in IPSC decay kinetics by around 1 week (compare Fig. 4 *A* with 4 *C*), suggesting that the turnover of GlyR channels and protein may be relatively slow. A general increase in mRNA for the AMPAR subunits was observed after Hearing onset, with the largest increase in the GluA4 subunit, which has the fastest kinetics (Fig. 4 *D*). This was well correlated with the acceleration in the EPSC kinetics (Figs 3 *B* and 4 *A*).

### Susceptibility to AT increases with maturation

**Figure 5:**
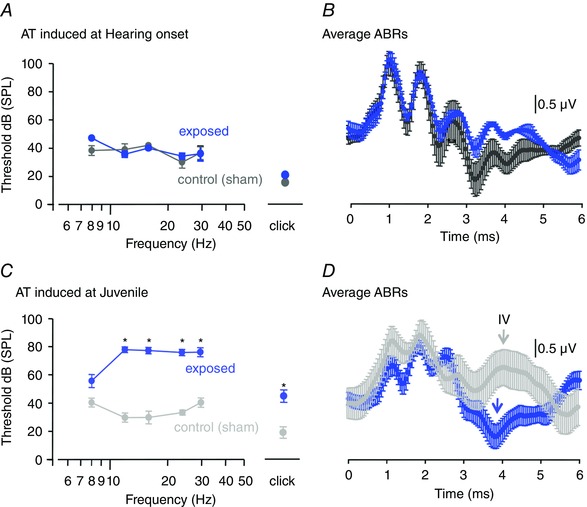
**Acoustic trauma (AT) caused little hearing loss at Hearing onset, but raised thresholds in mice after Hearing onset** *A*, AT was induced at Hearing onset and the ABR measured 1 week later. Control (sham) ABR thresholds (mean ± SEM, grey, *n* = 3) are plotted alongside ABR thresholds after induction of AT (blue, *n* = 3). *B*, average ABR traces (±SEM) for control (grey) and AT‐exposed (blue) mice at Hearing onset. *C*, AT was induced in Juvenile mice and ABRs were measured 1 week later. The ABRs showed significantly elevated thresholds; Control (grey, *n* = 10) and after AT (blue, *n* = 8). Significance is by *P < 0.0001 in each case (unpaired *t* test for clicks and two‐way ANOVA for the tone frequencies). *D*, average ABR traces (± SEM) for control (grey) and AT‐exposed (blue) Juvenile mice; ABR traces are for 24kHz tones at 94 dB SPL.

The above results provide a baseline from which experience‐dependent plasticity can be explored in the LSO following induction of an AT. AT was induced in anaesthetized CBA/Ca mice on exposure to 110 dB SPL broadband noise for 2 h. ABRs were measured before and one week (±1 day) after exposure. When acoustic over‐exposure was delivered at Hearing onset (P12–15), where ABR thresholds were still relatively high (Fig. 2 *B*), AT did not affect ABR waveforms or thresholds across a range of sound frequencies, as measured 1 week after exposure (Fig. 5 *A*, *B*). By contrast, when Juveniles (P17–23) were exposed to the same level of sound, ABR thresholds remained elevated by ∼40 dB, 1 week after the AT insult (i.e. mice now aged P23–30, Fig. 5 *C*). The characteristic ABR waveform was also altered, so that wave IV, reflecting the collective output activity of the SOC, was reduced in amplitude or absent (control: 0.97 ± 0.53 μV *vs*. AT: −1.13 ± 0.32 μV, unpaired *t* test, *P* = 0.004; Fig. 5 *D*). The physiological changes induced in the brain by exposure to loud sounds were investigated by comparing synaptic responses from naive and AT‐exposed mice using patch‐clamp recording from LSO neurons in *in vitro* brain slices.

### AT selectively prolonged EPSC decays by decreasing GluA4 and increasing GluA1 expression

**Figure 6:**
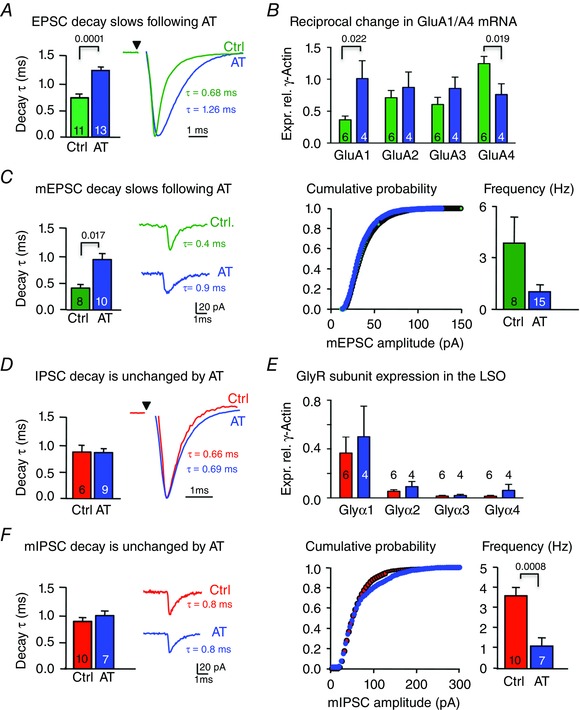
**AT prolongs EPSC decay time and increases GluR4 mRNA but has no effect on the IPSC time‐course** EPSCs are colour coded green, IPSCs in red and data from an AT exposed mouse are in blue; mean ± SEM, *n* values are indicated in the bar graphs. Synaptic currents were recorded from neurons held at −70 mV. *A*, evoked LSO EPSC decay time‐constants are plotted for control CBA/Ca mice (green) and in data from mice exposed to AT (blue) 1 week earlier. Right inset shows superimposed average EPSCs (*n* = 10 traces) from a control and AT‐exposed mouse. *B*, qRT‐PCR for GluA1–4 relative to γ‐actin, measured from control (green) and AT‐exposed (blue) mice. AT increased LSO GluA1 and decreased GluA4. *C*, miniature spontaneous EPSCs (average of 20 events, recorded in the presence of 0.5 μm TTX) showed the same slowing of the decay τ to the evoked EPSC. AT caused no change in the mEPSC amplitude distribution or mEPSC frequency. *D*, evoked LSO IPSC decay τ values are plotted for control (red) mice and from mice exposed to AT (blue). Right inset shows superimposed average IPSCs (*n* = 10 traces) from a control and AT‐exposed mouse. *E*, qRT‐PCR for Glyα1–4 relative to γ‐actin, measured from control (red) and AT‐exposed (blue) mice. LSOs exhibited no significant changes in GlyR subunits on AT exposure. *F*, miniature spontaneous IPSCs (average of 20 events, recorded in the presence of 0.5 μm TTX) and mIPSC amplitude were also unaffected by AT, but did reduce mIPSC frequency. Amplitude distributions are shown as cumulative distribution and were not significantly different (Kolmogorov–Smirnov test). Significance was determined by unpaired *t* tests for graphs *A*, *C*, *D* and *F*, and one‐way ANOVA for *B* and *E*, with *P* values as indicated.

Following induction of AT at P17–23, the mice were sacrificed 1 week later and *in vitro* brainstem slices were prepared so that excitatory and inhibitory synaptic responses could be examined in LSO principal neurons. No significant changes were observed in the amplitude of electrically evoked AMPAR‐mediated EPSCs at holding potentials of –70 mV following AT (controls: 7.11 ± 2.7 nS, *n* = 9; noise exposed: 6.4 ± 0.8 nS, *n* = 11, *P* = 0.81). Rise times for AMPAR‐EPSCs under control (*n* = 11) and following AT (*n* = 11) were also unchanged (values were 0.54 ± 0.05 and 0.55 ± 0.06 ms, respectively, *P* = 0.923). However, EPSC decay times were significantly prolonged after AT (Fig. 6 *A*; controls: 0.69 ± 0.05 ms, green traces, *n* = 11; noise exposed: 1.23 ± 0.05 ms, blue traces, *n* = 13, *P* < 0.001).

Consistent with this observation, qRT‐PCR of LSO tissue from mice treated identically to those used for the brain slice experiments showed two important changes in AMPAR mRNA expression: GluA1 mRNA expression increased (*P* = 0.022) while the GluA4 subunit expression decreased after AT (Fig. 6 *B*) (*P* = 0.019). These mRNA levels and EPSC kinetics are consistent with recordings from other native neuron types having slow AMPAR kinetics (deactivation decay time‐constants around 2–3 ms) when expressing a high abundance of GluA1 subunits (Yang *et al*. 2011) or other neurons with fast AMPAR kinetics (deactivation kinetics of < 1 ms) in neurons expressing a high proportion of GluA4 (see table 1 of Geiger *et al*. 1995).

A postsynaptic mechanism for the change in EPSC time‐course was supported by the analysis of mEPSCs (Fig. 6 *C*). The kinetics of mEPSCs were significantly prolonged after induction of AT; decay tau increased from 0.4 ± 0.06 ms (green traces, *n* = 8 cells) in control mice to 0.9 ± 0.1 ms (blue traces, *n* = 10) in AT‐exposed mice (*P* = 0.0017). AT had no significant effect on the frequency of mEPSCs in control and AT‐exposed mice (3.9 ± 1 Hz, *n* = 8; and 1.1 ± 0.4 Hz, *n* = 15, respectively, *P* = 0.12, see Fig. 6 *C*). mEPSC amplitude was also unaffected (control mEPSCs 26 ± 2.3 pA, *n* = 8; and AT‐exposed 26 ± 1.2 pA, *n* = 13, *P* = 0.95; HP=−80mV).

### AT does not change IPSC kinetics

We and others have previously shown that the role of GABA_A_ receptors in mediating the IPSC declines with maturation of the LSO, so that there is little or no GABA_A_ IPSC in Young adult mice and the IPSC is mediated by GlyRs. Inhibitory inputs to the LSO exhibit activity‐dependent synaptic refinement during development (Sanes & Friauf, 2000; Kandler & Gillespie, 2005; Kandler *et al*. 2009; Kramer *et al*. 2014). Here we show that glycinergic IPSCs in mature animals do not exhibit activity‐dependent plasticity of their subunit composition following AT. Rise times of LSO IPSCs for control and AT‐exposed mice were not significantly different (0.39 ± 0.05 ms, *n* = 5; and 0.36 ± 0.04 ms, *n* = 5, respectively, *P* = 0.7). Decay times and conductance magnitudes were also indistinguishable (Fig. 6 *D*; control tau: 0.87 ± 0.10 ms, *n* = 6; AT‐exposed tau: 0.84 ± 0.06 ms, *n* = 9, *P* = 0.83; control conductance, 7.2 ± 2 nS, *n* = 5; AT‐exposed conductance: 9.7 ± 4 nS, *n* = 6, *P* = 0.57). In accordance with these findings there was no change in mRNA for GlyR subunits (Fig. 6 *E*: Glyα1, *P* = 0.97; Glyα2, *P* = 0.69; Glyα3, *P* = 0.62; Glyα4, *P* = 0.63) or in mIPSC kinetics (Fig. 6 *F*, *P* = 0.466). mIPSC amplitude was unaffected whereas mIPSC frequency was reduced following AT (control amplitude: 39 ± 2.8 pA, *n* = 9; AT‐exposed: 31 ± 1.5 pA, *n* = 4, *P* = 0.1, HP=−80mV; control frequency: 3.6 ± 0.4 Hz, *n* = 10; AT = 1.1 ± 0.3 Hz, *n* = 7, *P* = 0.0008) as shown in Fig. 6 *F*. Hence, AT specifically alters EPSC kinetics, but has no effect on IPSC kinetics. The reduction in frequency of miniature synaptic responses implies a possible presynaptic reduction in release probability, but this was only significant at inhibitory synapses and the overall excitatory/inhibitory balance was similar, at around 1 Hz after AT (Fig. 6 *C vs*. 6 *F*). Further study of presynaptic changes will require examination of unitary evoked inputs and is a topic for future studies. We conclude that AT induces slowing of the decay kinetics in both evoked and miniature EPSCs, consistent with a reduced dominance of fast‐gating GluA4 in the subunit composition of synaptic AMPAR at the excitatory synapse.

### NMDAR‐EPSCs accelerate with development, but their decay is unchanged by AT

**Figure 7:**
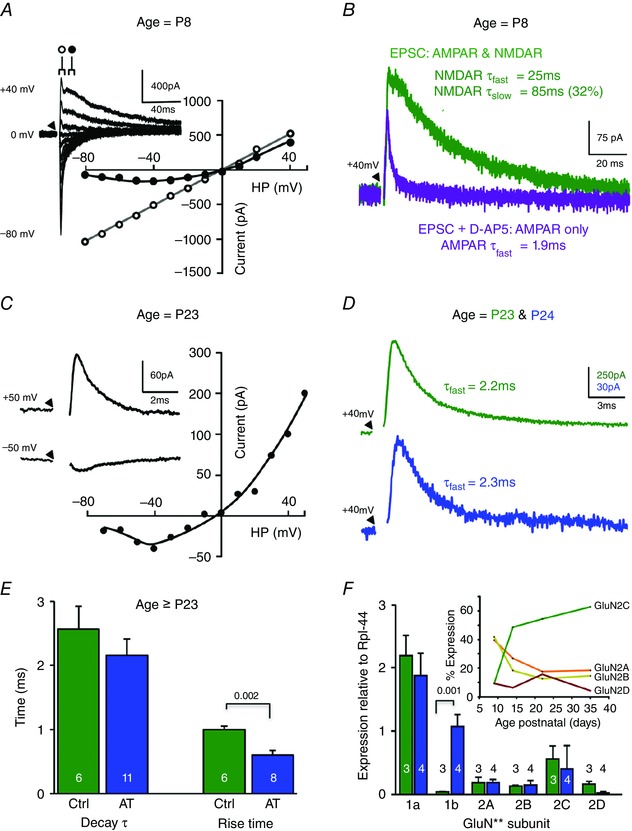
**LSO NMDAR‐mediated EPSC decay time accelerates during development** *A*, evoked EPSC *I–V* relationship from a pre‐hearing (P8) LSO principal neuron (in the presence of 10 μm bicuculline and 1 μm strychnine). Open circles indicate the latency at which the *I–V* of the fast AMPAR‐EPSC is measured, closed circles show the latency for the *I–V* of the NMDAR‐EPSC. Inset: superimposed average EPSC traces for each holding potential (HP) from −80 to +40 mV. Throughout, the stimulus is indicated by a triangle, with artifacts being removed for clarity. *B*, in a Pre‐hearing mouse (P8) an LSO neuron held at a potential of +40 mV shows the evoked EPSC before (green trace) and after perfusion of 20 μm d‐AP5 in the same cell. The fast AMPAR‐mediated response remains in the presence of AP5 (purple trace) and confirms that the slow EPSC is mediated by NMDAR. The EPSC time‐constants are indicated by the respective trace. *C*, in a mature mouse (P23) the EPSC from an LSO neuron is recorded in the presence of 10 μm bicuculline, 1 μm strychnine and 10 μm NBQX to isolate the NMDAR component. Inset: average traces from HP of +50 and −50 mV. Note the voltage‐dependence of the EPSC and the fast decay time‐course of this NMDAR‐mediated EPSC. *D*, NMDAR‐EPSCs from Control (green) and AT‐exposed (blue) mice (>P23) show similar fast decay time‐courses at +40 mV HP. *E*, bar chart showing mean NMDAR‐EPSC decay τ and rise time for control and AT‐exposed mice, respectively. *F*, relative expression of GluN1 and GluN2 subunit mRNA in the LSO from control (green) and following AT exposure (blue). GluN1b mRNA at P35 was significantly increased following AT. AT in mature mice increased GluN1b mRNA and decreased the NMDAR‐EPSC rise time, but had no impact on decay time‐course. Inset: developmental profile of GluN2 subunit mRNA expression shows GluN2C mRNA levels increase and dominate over maturation. Statistical comparison was performed using a *t* test in *E* and one‐way ANOVA in *F* with *P* values as indicated.

It is well established that glutamatergic, excitatory synaptic responses are generally composed of a fast, AMPAR‐mediated EPSC and a slower, voltage‐dependent EPSC mediated by NMDAR. Similar results have been observed in the immature MNTB (Steinert *et al*. 2010) and LSO (Case & Gillespie, 2011) and as shown here in the LSO from Pre‐hearing mice (Fig. 7 *A*). This NMDAR‐mediated EPSC was blocked by 20 μm d‐AP5 (Fig. 7 *B*) and decayed with a double exponential comprising a tau_fast_ of 44.8 ± 5.4 ms and a tau_slow_ of 115 ± 17.1 ms (*n* = 8). The tau_slow_ contributed 34% of the total amplitude. In Post‐hearing mice the NMDAR‐EPSC rapidly ran down on dialysis during whole cell patch recording, so that peak amplitude had decayed to half initial values in 43.4 ± 1.8 s (*n* = 5), which made detailed pharmacological studies difficult and may explain why this response has not been observed previously. Nevertheless, the voltage dependence of the NMDAR‐EPSC was maintained (Fig. 7 *C*) and the acceleration in decay kinetics was dramatic, with time‐constants of tau_fast_ of 2.6 ± 0.4 ms and tau_slow_ of 28.8 ± 8.7 ms (*n* = 6) in animals older than P23. The tau_slow_ was small, contributing on average only 8.7% of the combined current amplitude and was on the limits of detectability. Following acoustic trauma only tau_fast_ decay could be reliably observed and fit by a single exponential, which was not significantly different from control (tau_fast_ = 2.2 ± 0.3 ms, *n* = 11, *P* = 0.351; Fig. 7 *D*, *E*). The rise time for the NMDAR‐EPSC under control was 0.99 ± 0.06 ms (*n* = 6) and this accelerated to 0.60 ± 0.07 ms (*n* = 8) following AT (*P* = 0.002, Fig. 7 *E*). Examination of NMDAR subunit mRNA levels showed one significant change following exposure to AT: there was an increase in GluN1b mRNA (*P* = 0.001), while GluN1a and the GluN2 subunits were unchanged (Fig. 7 *F*). The inset to Fig. 7 *F* shows that the developmental maturation of expression of GluN2 subunits in the LSO is similar to previous reports from the MNTB, where GluN2C dominates on maturation (Steinert *et al*. 2010). We conclude that NMDAR‐EPSCs are present in the LSO, and in mature mice have remarkably fast kinetics (similar to those of AMPAR‐mediated EPSCs in some brain areas). It seems likely that an NMDAR‐mediated conductance with such fast kinetics may have been missed in the past, either because many recordings were made from immature animals, and/or because it merged with the AMPAR‐mediated EPSC and was not recognized or because it runs down so quickly on whole‐cell dialysis. We conclude that AT had little influence on the decay of NMDAR‐EPSCs, although the induced faster rise time which correlated with higher levels of GluN1b expression will be a topic for further study.

### AT disrupts LSO integration of excitation and inhibition

**Figure 8:**
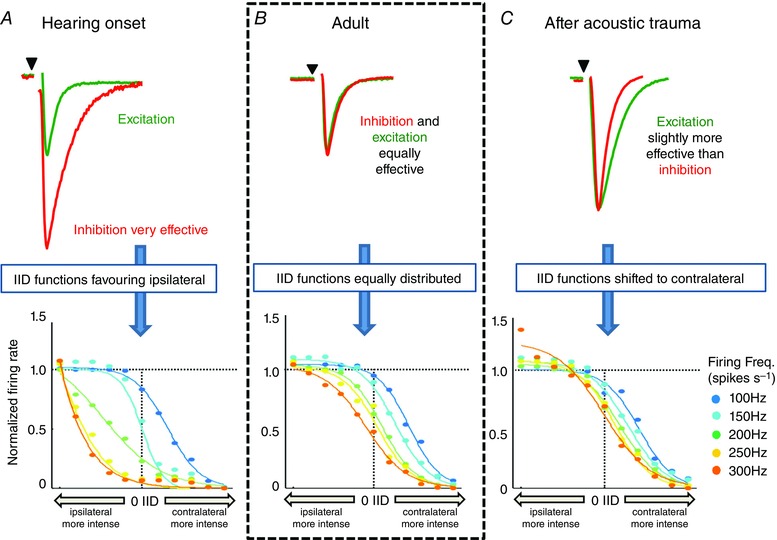
**Modelling shows that convergence of EPSC and IPSC kinetics is required for maturation of IID functions** The relative time‐course of excitatory (green) and inhibitory (red) synaptic inputs to the LSO is shown for the three conditions: Hearing onset, Adult and following acoustic trauma. In the lower graphs the LSO model output is shown normalized to the input firing rate (corresponding to sound intensity) plotted against the IID across a range of firing frequencies from 100 to 300 Hz (colour coded as indicated on the right: 100 Hz, blue; 300 Hz, orange). *A*, at Hearing onset, inhibitory synaptic currents in the LSO have slow kinetics and large amplitudes. This makes inhibition more effective than excitation and shifts IID functions to the left for higher input frequencies. *B*, balanced amplitudes and kinetics of EPSCs and IPSCs result in a normal distribution of IID functions in the mature LSO. *C*, AT causes a deceleration of the excitatory synaptic currents, increasing the effectiveness of excitation and leading to a rightward shift of the IID functions.

Neurons in the LSO are the first to integrate information about IID (for a review see Tollin, 2003). LSO output can be characterized by the slope and half‐activation of its IID functions (Park *et al*. 2004; Karcz *et al*. 2011), and the effectiveness of this tuning can be quantified with Fisher information (see Methods). Mature IIDs are achieved by integrating ipsilateral excitatory and contralateral inhibitory inputs, which must converge in temporal register (Joris & Yin, 1995; Tollin, 2003). Here we show that the kinetics of IPSCs and EPSCs converge to near identical values during development, reaching sub‐millisecond time‐constants in Young adult mice (Figs 3 *A* and 4 *A*). Incorporating the synaptic current data into a computational model of LSO neurons, and optimizing the spiking threshold as a free parameter to produce optimal tuning curves showed that IID tuning becomes more effective and precise as development proceeds (see Methods). Poor tuning was characterized by shallow tuning curves, and to a lesser extent by differences in variability (not illustrated). The slow time‐course of inhibition at Hearing onset essentially renders IID detection impossible during high rate synaptic activity (Fig. 8 *A*, *B*), while at modest stimulus levels, IID tuning is comparable to the adult performance. In contrast, adult tuning is effective over a wide range of input firing rates, with curves shifting progressively towards more negative IIDs, and consistent with experimental observations (Tsai *et al*. 2010).

Simulated IID functions using synaptic parameters matched to those after AT were shifted to the right, particularly for strong stimuli (Fig. 8 *C*, contralateral stimulus more intense). IID discrimination performance for weak stimuli (low sound intensity) was essentially unchanged by AT, but IID sensitivity for strong stimuli was predicted to be poor due to the imbalance between excitatory and inhibitory inputs; inhibition failed to effectively suppress the stronger excitation caused by longer‐lasting EPSCs (EPSCs: 1.23 ± 0.05 ms, *n* = 13; IPSCs: 0.83 ± 0.06 ms, *n* = 9, *P* < 0.001).

What are the implications of this result for binaural IID detection? We observed that ABR thresholds are strongly elevated following AT (Fig. 5 *C*), suggesting that under normal conditions, average neural activity in the auditory brainstem will be reduced compared to normal adults. The simulations show that IID detection can be fully effective for weaker activity levels. Therefore, we expect IID tuning to be largely intact for the physiological range of sound‐evoked activity in noise‐exposed animals. In fact, Fisher information was slightly increased at low stimulus frequencies up to about 100 Hz in simulations within the AT group, compared to normal adults (data not shown). This improvement, which was caused by a suppressed response variability due to stronger excitatory drive, was however achieved at the expense of performance at higher firing frequencies (i.e. sound intensity). Overall, these results suggest that the slowing of excitatory synaptic transmission following acoustic trauma has a detrimental effect on IID tuning for higher rates of neural activity.

### Recovery of the EPSC time‐course and ABR waveforms

**Figure 9:**
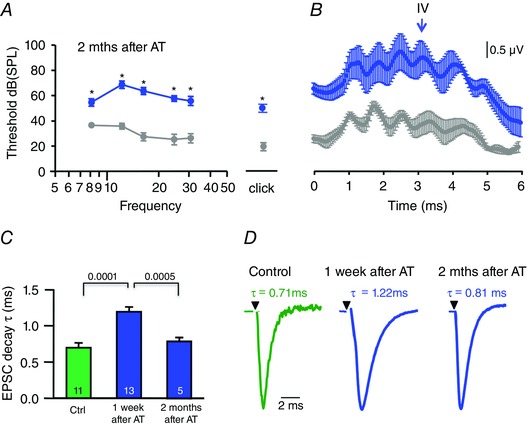
**The AT‐induced plasticity in the LSO EPSC is reversible** *A*, plot showing average ABR thresholds at 8–30 kHz (mean ± SEM) and for a click stimulus. Thresholds remained elevated 2 months after AT (blue) at around P90, compared to similar aged mice which received no AT (grey). *B*, averaged ABRs (24 kHz, tone pip at 94 dB SPL, mean ± SEM) for the same data set, showing that wave IV is present in control and on recovery from AT by around P90. *C*, AMPAR‐EPSC decay time‐constants (τ) were measured from the same mice as in *A* and *B*. Bar chart shows EPSC decay τ (mean ± SEM) from unexposed mice (control, green) and mice that received AT 1 week earlier (blue, *n* = 13) or months earlier (blue, *n* = 5). *D*, representative AMPAR‐EPSC averages (*n* = 20) for each group; the slowed EPSC decay τ had recovered to control values 2 months after AT induction. Significance was assessed by two‐way ANOVA for tone frequencies (*P* < 0.0008) and unpaired *t* test for clicks in *A* (*P* = 0.002) and by one‐way ANOVA in *C* (*P* values as indicated).

Finally we investigated whether the effects of AT were permanent. We measured ABRs and excitatory synaptic inputs to the LSO 2 months after trauma in P90–96 mice. We observed a partial recovery of the ABR thresholds (thresholds were elevated by 30 dB compared to control) and a full recovery of the ABR waveforms (Fig. 9 *A*). Control, untreated mice and noise‐exposed animals ∼3 months old showed similar ABR responses with the characteristic ABR peaks I–V (Fig. 9 *B*). Wave IV of the ABR which was absent (or reduced) 1 week after AT recovered and the EPSC decay time recovered to control levels, reaching similar values to those found in naïve mice (EPSC_tau_: 0.8 ± 0.04 ms, *n* = 5, Fig. 9 *C*, *D*). This suggests that slow, delayed excitation in the LSO could contribute to altered ABR wave IV and the shift in IID functions.

## Discussion

We first determined the maturation of evoked EPSC and IPSC kinetics in the LSO during development from Pre‐hearing (P7–P9) to Young adult (P36) mice. The decay kinetics of the synaptic currents both accelerate and converge to precisely matched sub‐millisecond values by P17 and are maintained into adulthood. We then asked how exposure to damaging levels of sound (AT) might influence the synaptic kinetics in the LSO. We found using Young adult mice that the evoked and miniature AMPAR‐mediated EPSC decay was slowed after the insult; this was mirrored by a rise in GluA1 and a decrease in GluA4 mRNA levels in the LSO. AT had no influence on the glycinergic IPSC kinetics, so there was now a mismatch in the synaptic integration of the E/I inputs which modelling showed undermines the accuracy of the IID computation. Evoked NMDAR‐EPSCs also showed dramatic acceleration with developmental maturation, with a time‐constant of over 40 ms in Pre‐hearing mice, while in mature mice the NMDAR‐EPSC decayed with a time‐constant of around 2 ms. Although AT caused an increase in expression of GluN1b, we found no change in the decay of the evoked NMDAR‐EPSC.

This study set out to investigate whether acoustic trauma caused long‐term changes in central auditory processing (IID) by examining changes in the postsynaptic expression of receptor subunits. The convergence in the IPSC/EPSC time‐course occurred simultaneously with ABR maturation and was consistent with simulations of IID functions across the physiological range. AT disrupted both EPSC/IPSC integration and IID function in the LSO and was consistent with a change in the levels of mRNA for glutamate receptor subunits, while IPSC kinetics and GlyR subunit mRNA were unaltered.

### Development of fast inhibitory inputs

The term ‘hearing onset’ is often used rather loosely to refer to the time at which the auditory canal opens; in rats and mice this is around P12, although it should be noted that with high sound intensity bone conduction permits detection of ABRs in neonatal rats as young as P7–P8 (Geal‐Dor *et al*. 1993). Developmental changes in the IPSC prior to opening of the auditory canal are well documented, as a refinement in the number of inhibitory inputs (Sanes & Friauf, 2000; Kandler *et al*. 2009) and changes in the extent of axonal arbors (Sanes & Siverls, 1991). In parallel, there is a developmental shift from mixed GABA/glycinergic IPSCs (Lim *et al*. 2000; Smith *et al*. 2000) towards glycinergic transmission which becomes dominant in the adult LSO (Kotak *et al*. 1998; Nabekura *et al*. 2004; Lohrke *et al*. 2005; Kim & Kandler, 2010). After Hearing onset there is further acceleration of the IPSC kinetics by P18 (Walcher *et al*. 2011), which we confirm here, and show that the fast kinetics is maintained to P35. Glycinergic synaptic currents accelerate during development and this follows changes in the expression of GlyR subunits (GlyRs) from GlyRα2 to GlyRα1 in mature animals (Becker *et al*. 1988; Takahashi *et al*. 1992). GlyRα1‐containing channels display faster kinetics than other GlyR subunits (Lynch, 2009) and expression of GlyRα1 mRNA increases in the LSO after Hearing onset (Piechotta *et al*. 2001). qRT‐PCR confirmed a switch from GlyRα2 to GlyRα1 mRNA from around Hearing onset with no significant change in other GlyR subunits.

IPSC peak conductance declined up to Hearing onset, consistent with anatomical pruning of MNTB axon terminals in the LSO (Sanes & Siverls, 1991; Sanes *et al*. 1992; Kandler *et al*. 2009). Fast glycinergic transmission typically has IPSC decay time‐constants of around 1 ms (Stuart & Redman, 1990; Jonas *et al*. 1998; Smith *et al*. 2000; Legendre, 2001; Awatramani *et al*. 2004; Magnusson *et al*. 2005; Bowery & Smart, 2006; Lu *et al*. 2008). Co‐release of GABA with glycine is commonly observed at inhibitory synapses (Jonas *et al*. 1998; Lim *et al*. 2000; Kim & Kandler, 2003; Fischl & Burger, 2014) and can accelerate each other's kinetics (Lu *et al*. 2008) although in the LSO no evidence for a significant GABAergic component to the IPSC was observed in mice at ages after weaning (Sterenborg *et al*. 2010). Our observations concur with the general developmental time‐course of GlyRs observed previously in the rat (Friauf *et al*. 1997). The molecular basis for the transition to fast glycinergic IPSCs is unclear, as the acceleration continues after Hearing onset when GlyR1 is already highly expressed. Our observations show that similar IPSC and EPSC time‐courses contribute to effective integration of binaural information, as also suggested from other studies (Reed & Blum, 1990). Although IPSC kinetics are not affected by AT, a decrease in the frequency of spontaneous synaptic events following AT suggests an additional presynaptic mechanism, which will require further investigation. Disruption of hearing (through cochlear ablation, age, ear‐plugging or noise exposure) is generally associated with decreased levels of inhibition (Leao *et al*. 2004; Vale *et al*. 2004; Kotak *et al*. 2005) and GlyRs are down‐regulated in the cochlear nucleus after cochlear ablation (Sato *et al*. 2000; Asako *et al*. 2005). Ear‐plugging down‐regulated Glyα1 in the cochlear nucleus and increased GluA3 (Whiting *et al*. 2009), although in the LSO neither IPSC time‐course nor GlyR subunit mRNA was significantly altered by AT.

### Fast AMPAR‐mediated excitatory transmission in the LSO

AMPAR‐mediated EPSCs with fast kinetics are widely expressed in the auditory pathway, driving high‐fidelity neurotransmission in birds and mammals (Raman & Trussell, 1992; Zhang & Trussell, 1994; Barnes‐Davies & Forsythe, 1995; Golding *et al*. 1995; Otis *et al*. 1995; Gardner *et al*. 1999, 2001; Trussell, 1999; Joshi *et al*. 2004; Koike‐Tani *et al*. 2005, 2008; Steinert *et al*. 2010). Expression studies show that EPSCs exhibiting rapid kinetics are associated with GluA4‐containing AMPARs (Mosbacher *et al*. 1994; Geiger *et al*. 1995; Lambolez *et al*. 1996), which are broadly expressed in the auditory brainstem (Rubio & Wenthold, 1999; Yang *et al*. 2011), and these receptor subunits are predominantly flop spiced variants (Schmid *et al*. 2001). Although the EPSC decay kinetics in the MNTB (∼0.4 ms) (Taschenberger & von Gersdorff, 2000; Futai *et al*. 2001; Fernandez‐Chacon *et al*. 2004; Postlethwaite *et al*. 2007) are somewhat faster than in the LSO (∼0.7 ms), the prevalence of GluA4 mRNA in the LSO is consistent with the observed fast EPSC decay kinetics. The crucial contribution of GluA4 subunits to fast EPSCs has been demonstrated in the MNTB, where synaptic responses from the calyx of Held were slower and smaller in GluA4 knockout mice than in wild‐type animals (Yang *et al*. 2011). Here we correlated the EPSC kinetics with mRNA levels of the receptor subunits during development and after exposure to AT. Although changes in mRNA levels were significant, it is worth noting that the relationship between mRNA concentration and protein expression is not linear and many factors may also influence the translation into protein.

AMPAR‐subtype remodelling occurs after repetitive synaptic stimulation, sensory deprivation, drug addiction and pathological conditions, such as epilepsy (Grooms *et al*. 2000; Liu & Cull‐Candy, 2000; Clem & Barth, 2006; Goel *et al*. 2011). Here, high levels of acoustic stimulation reduce expression of GluA4 mRNA and slow AMPAR‐mediated EPSCs. A similar molecular mechanism of plasticity has been implicated in the classical eye‐blink conditioning (Zheng & Keifer, 2009). Our modelling shows that this increased excitatory charge transfer boosts the compromised excitatory pathway, presumably enabling audible stimuli to provide a stronger excitatory input than in a normal animal. This slower time‐course is a homeostatic process to rescue binaural function following AT, rather than a ‘reversal’ to a less mature state. This hypothesis is consistent with dynamic regulation of glutamate receptors during plasticity at other synapses, where GluA1 up‐regulation is clearly associated with a persistent decrease in activity (Granger *et al*. 2011). GluA4 is essential for rapid kinetics (Raman & Trussell, 1992; Yang *et al*. 2011) and for homeostatic control of excitatory synaptic transmission in the mature brain (Wierenga *et al*. 2005). AT reduces auditory firing rates (for a given SPL) due to the peripheral damage, and the increased excitatory drive observed in the LSO acts to enhance and compensate for this; therefore, IID detection is still (largely) intact at low firing rates. Overall the modelling shows that increased spontaneous firing following AT maintains binaural function in the LSO, in spite of peripheral hearing loss.

### NMDAR‐mediated EPSCs exhibit extreme acceleration on maturation of the LSO

As a consequence of defining the excitatory receptor subtypes of the ipsilateral projection to the LSO in Pre‐hearing mice, we observed the classical dual‐component EPSC (Forsythe & Westbrook, 1988) with a fast AMPAR‐mediated EPSC and a slow voltage‐dependent NMDAR‐mediated response. Over the next 2 weeks of development this d‐AP5‐sensitive EPSC accelerates to have a decay time‐constant of around 2.5 ms at +40 mV in the presence of [Mg^2+^]_o_. This contrasts with NMDAR‐EPSCs in immature animals which generally have decay time‐constants in the range 30–200 ms. Such rapid NMDAR‐mediated EPSCs have not been reported previously, although developmental acceleration of NMDAR‐EPSCs has been previously characterized in the MNTB (Steinert *et al*. 2010) where decay time‐constants accelerate to around 15 ms in mature mice and rats. Moderate acceleration of NMDAR kinetics during maturation is a common theme in many areas of the brain; hippocampus CA1 synapses have decays of around 100 ms (Kirson & Yaari, 1996; Rauner & Kohr, 2011) while at thalamo‐cortical synapses NMDAR‐EPSCs accelerate to around 50 ms at P27 (Barth & Malenka, 2001) and in cerebellar granule cells by P21–P40 the dominant fast decay tau is around 30 ms, although a contribution from a slow tau of around 300 ms continues (Cathala *et al*. 2000). This developmental acceleration is not universal and will depend on the particular subunits expressed; for example, in the rat nucleus accumbens, decay time‐constants of around 200 ms are stable from Juvenile to adult (Kasanetz & Manzoni, 2009). The acceleration observed here in the LSO is the most extreme yet observed and brings the NMDAR‐EPSC time‐course close to those of GluA1‐dominated AMPARs. NMDAR subunit mRNA showed similar changes to those published for the MNTB (Steinert *et al*. 2010) with adult dominance of GluN2C. The only significant change in NMDAR subunit mRNA following AT was an increase in GluN1b message relative to GluN1a. Although AT had little or no effect on the fast NMDAR‐EPSC decay, further investigation is required to understand the contribution of this EPSC to auditory processing in the LSO.

### Integration of excitation and inhibition encodes interaural intensity differences

Similar kinetics of excitatory and inhibitory transmission underlies encoding of mature IID functions and detection of a sound source. Mismatched synaptic kinetics shift the IID functions and lower performance (Pollak, 1988; Park *et al*. 1996, 1997, 2004; Klug *et al*. 2000; Tollin, 2003). For example, the IID population code in the LSO is skewed towards the excitatory ear in young (P12–17) animals (Sanes & Rubel, 1988) and also in animals lacking the potassium channel subunit, Kv1.1 (Karcz *et al*. 2011). These two cases suggest that inhibition is more effective than excitation. This change in efficacy can be mediated either by differences in thresholds (Sanes & Rubel, 1988; Tsai *et al*. 2010) or by slower and temporally dispersed inhibition (Karcz *et al*. 2011). Using the slow IPSC kinetics (from the young animals), simulation of IID functions supports the notion that this provides tonic inhibition, skewing IIDs to the excitatory ear (Fig. 8).

Our data indicate that the changes in LSO EPSCs after AT follow a similar principle, causing IID functions to be skewed to the ‘inhibitory ear’, as EPSC kinetics are slowed whereas inhibition remains fast. In this scenario, excitation outlasts inhibition and is therefore more effective (opposite to the above situation) and IID function is dominated by contralateral auditory space. IID discrimination was also enhanced for low spike rates, preserving function following peripheral damage. For stronger stimuli, shallow IID tuning curves reduce discrimination because excess excitation is not balanced by inhibition, highlighting the importance of carefully balanced timing, kinetics and magnitude of synaptic excitation and inhibition for normal LSO function. Similar conclusions were drawn from *in vivo* patch‐clamp recordings in the inferior colliculus of bats (Gittelman & Pollak, 2011).

Hearing deficits caused by AT, aging or genetic susceptibility are primarily considered from a peripheral and cochlea perspective, but changes and adaptations in central auditory processing can mitigate or compound the peripheral auditory deficit (Kujawa & Liberman, 2009; Pilati *et al*. 2012). We conclude that hearing damage causes changes in the subunit composition of synaptic AMPARs, with long‐term consequences for synaptic integration, but which are resolved by 2 months. Together with other forms of plasticity, this compensates IID for peripheral auditory damage.

## Additional information

### Competing interests

The authors declare no competing financial interests.

### Author contributions

Electrophysiological experiments were predominantly carried out by N.P., with additional data by D.M.L. on NMDAR‐EPSCs and qRT‐PCR by H.S. O.U. and I.D.F. obtained funding with initial ideas further developed and supervised by N.P., C.K.S. and I.D.F. M.H.H. conducted the modelling. N.P., D.M.L. and I.D.F. wrote the manuscript with input from all authors.

### Funding

This work was funded by the Wellcome Trust, the Medical Research Council, and Action on Hearing Loss and Rosetrees Trust. M.H.H. was supported by an MRC Career Development Award (G0900425).
